# Development and Validation of a UAV Based System for Air Pollution Measurements

**DOI:** 10.3390/s16122202

**Published:** 2016-12-21

**Authors:** Tommaso Francesco Villa, Farhad Salimi, Kye Morton, Lidia Morawska, Felipe Gonzalez

**Affiliations:** 1 International Laboratory for Air Quality and Health (ILAQH), Queensland University of Technology (QUT), 2 George St, Brisbane QLD 4000, Australia; tf.villa@hdr.qut.edu.au; 2 Menzies Institute for Medical Research, University of Tasmania, Hobart, Tasmania 7000, Australia; farhad.salimi@utas.edu.au; 3 Australian Research Centre for Aerospace Automation (ARCAA), Queensland University of Technology (QUT), 2 George St, Brisbane QLD 4000, Australia; kye.morton@hdr.qut.edu.au (K.M.); felipe.gonzalez@qut.edu.au (F.G.)

**Keywords:** UAV remote gas sensing, downwash effect, air quality, hexacopter, optical sensor, air pollution, particle number concentration monitor

## Abstract

Air quality data collection near pollution sources is difficult, particularly when sites are complex, have physical barriers, or are themselves moving. Small Unmanned Aerial Vehicles (UAVs) offer new approaches to air pollution and atmospheric studies. However, there are a number of critical design decisions which need to be made to enable representative data collection, in particular the location of the air sampler or air sensor intake. The aim of this research was to establish the best mounting point for four gas sensors and a Particle Number Concentration (PNC) monitor, onboard a hexacopter, so to develop a UAV system capable of measuring point source emissions. The research included two different tests: (1) evaluate the air flow behavior of a hexacopter, its downwash and upwash effect, by measuring air speed along three axes to determine the location where the sensors should be mounted; (2) evaluate the use of gas sensors for CO_2_, CO, NO_2_ and NO, and the PNC monitor (DISCmini) to assess the efficiency and performance of the UAV based system by measuring emissions from a diesel engine. The air speed behavior map produced by test 1 shows the best mounting point for the sensors to be alongside the UAV. This position is less affected by the propeller downwash effect. Test 2 results demonstrated that the UAV propellers cause a dispersion effect shown by the decrease of gas and PN concentration measured in real time. A Linear Regression model was used to estimate how the sensor position, relative to the UAV center, affects pollutant concentration measurements when the propellers are turned on. This research establishes guidelines on how to develop a UAV system to measure point source emissions. Such research should be undertaken before any UAV system is developed for real world data collection.

## 1. Introduction

Unmanned Aerial Vehicles (UAVs), carrying onboard sensors, can be used to directly measure shipping emissions, emissions from industrial stacks or ground vehicles when it is too difficult or dangerous to use both manned aircrafts [1] and ground level stations [2]. However, accurate sampling of small plumes emitted by combustion sources such as trucks, petrol locomotives, ships and dredgers, industrial and even domestic chimneys demands appropriate location of the air sensor intakes onboard the UAV. Therefore, the use of UAVs for air pollution measurement, particularly at slow speeds or stationary flights, can only be effective if the location point of the air sensor intake is optimized, such that sampling of gaseous and particulate matter pollutants is done before the propellers of the UAV mix or disperse the plume. The air is deflected by the action of the UAV propellers in motion, when producing lift, and this is called the downwash and upwash effect. It is the transport of gas to the gas sensors that is the critical process, and accurate sampling relies on assessing the contribution of rotor disturbance to this process. 

Only two studies were found [3,4] to investigate the sensor mounting point onboard a multirotor UAV to measure air pollution. The first study designed three different experiments to identify the best location of a CO_2_ gas sensor intake onboard a quadrotor and how to bring the air to the sensor [3]. The three experiments included: an active system using an additional fan, a semi-active system using the airflow generated by the rotors, and a passive system, without any auxiliary device directing the airflow. Significant differences between the three different gas transport solutions were found and no system was capable of measuring the reference gas concentration (0.5% by volume of CO_2_). Overall, the authors [3] established that the position of the tube inlet strongly affected the gas measurement results and wind resistance caused by the enlarged drone.

The second study [4] investigated the airflow of a small quadcopter using a Computational Flow Dynamic (CFD) simulation and found that the maximum air speed was at the rotor perimeters and that the minimum was at the center of the UAV. The researchers proved this by attaching the UAV to a cardan joint to measure air speed above and below the platforms. Above the UAV center was the best mounting point. The paper failed, however, to provide information about the height of the cardan joint, ground effect, or mounting point along aside the UAV, which was a quadcopter (Parrot AR drone 2.0) for hobby use with both a limited payload (<100 g) and a flight endurance of less than 12 min with a zero-weight payload. 

Optimizing the sensor mounting location can improve how the pollutant samples emitted by a point source are represented. This research aimed to: (1) determine the best location for mounting a gas and PNC sensing payload intake onboard a hexacopter by measuring air speed along its three axes; (2) to validate the UAV based system capable of measuring CO_2_, CO, NO_2_ and NO gases and PNCs by measuring emissions from a diesel engine. 

## 2. Unmanned Airborne Plume Assessing System 

### 2.1. Overview

Hexacopter UAVs provide a larger payload capacity (>2 kgs payload), and more in-flight stability and maneuverability compared to quadrotors, making them suitable for UAV and air quality studies where the capability to carry different sensors and maintain an in-flight fixed position are needed [5]. However, a comprehensive study on sensor location on such platforms has not been done yet.

The system developed and evaluated in this research consists of a multi-rotor UAV, four gas sensors for CO, CO_2_, NO and NO_2_, a DISCmini (Portable PNC monitor, Testo AG, Lenzkirsch, Germany), temperature and humidity sensors as well as a real-time visualization interface. All sensors are integrated with an Arduino MEGA 2560 microcontroller board. 

### 2.2. System Architecture

**Figure 1:**
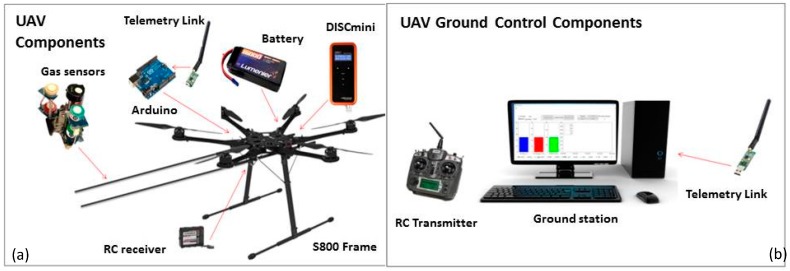
(**a**) Unmanned aerial vehicle (UAV) system components including gas sensors, DISCmini, Arduino MEGA 2560, battery and RC receiver; (**b**) UAV Ground Control Components for both manual (using RC transmitter) and autonomous (using PC) operations.

Figure 1 shows an overview of the system, the gas sensing payload and the PNC monitor, while the UAV system architecture is shown in Figure S1 in the Supplementary Material.

The system presented in Figure 1 comprises: (a) the UAV system components including DJI S800, DISCmini and gas sensors, Arduino board, telemetry, and RC receiver, and (b) Ground Control Components including RC transmitter for the pilot, and GSO (ground station + telemetry link). 

Figure S1 (Supplementary Material) shows UAV system hardware and software, including the ground control station, with the connection types between the different UAV components highlighted. 

The UAV pilot or the UAV ground station operator (GSO) can communicate with the UAV wirelessly, using a radio controller (RC) transmitter or a computer, respectively. The purple arrows show pilot and GSO inputs.

### 2.3. UAV

The UAV used in this research is a modified hexacopter S800 EVO manufactured by DJI (Shenzhen, China) [6]. The frame measures 800 mm in width and 320 mm in height and is made of composite materials. The built-in damping system allows the multi-rotor to be assembled without additional frames and dampers. The UAV weighs 3.7 kg with a maximum take-off weight of 8 kg. The S800 is designed to operate under the 20 kg all up weight (AUW) class of UAS (Unmanned Air System) as defined by the Civil Aviation Safety Australia (CASA) to reduce operation costs, and avoid being subject to the tighter CASA regulations for larger UAVs [7]. Different countries may have different aviation regulations which should be considered in the design of a UAV system. For example, in the US, the Federal Aviation Administration (FAA) considers small UAVs those with a weight less than 55 lb (25 kg) [8]. 

The UAV uses a 16,000 mAh LiPo 6 cell battery, which provides a hover time of approximately 20 min with no additional payload. However, the flight time with the payload used in this study has been calculated to be about 12–13 min. The hovering motor power consumption is 800 W operating with a minimum take-off weight. Motors run in conjunction with 38 cm × 13 cm propellers of 13 g each. 

### 2.4. Sensor Selection

Gas sensors are classified according to their operational principles with the most common being thermal, mass, electrochemical, potentiometric, amperometric, conductometric, and optical sensors [9,10]. PM sensors differ in terms of monitoring PM in the range of PM_10_ (mass concentration of particles with aerodynamic diameter <10 µm) and PM_2.5_ (<2.5 µm) and the ultrafine fraction of PM (<0.1 µm). Many “built in” devices, with a sensor incorporated as an integral part of the device itself, already exist for PM_10_ and PM_2.5_. However, limited options are available for PNC and size distribution monitoring devices and those existing include: the Nanotracer Monitor from Philips [11] and the Mini Diffusion Size Classifier (DISCmini) developed by the University of Applied Sciences, Windisch, Switzerland [12,13]. Both devices operate on similar principles, and the later was selected for this research.

The DISCmini is a portable instrument of relatively small dimensions (180 mm× 90 mm× 40 mm), low weight (640 gr, 780 gr including probe provided by the supplier) and long battery duration (8 h operative). It is used to measure the number of particles with diameters ranging between 10 and 500 nm, with a time resolution of 1 s (Supplementary Material, Table S1) and an experimentally tested response time of 7.3 s [14]. The DISCmini can measure a concentration range from about 10^3^ to over 10^6^ p/${\mathrm{cm}}^{3}$, based on the electrical charging of the aerosols. The sensor works by generating positive air ions in a corona discharge subsequently mixed with the aerosol. Measurement accuracy depends on the shape of the particle size distribution and number particle concentration; it is usually around 10%–15% compared to a reference condensation particle counter (CPC). Unlike the CPC or other larger instruments, the DISCmini does not need a liquid to grow and count particles and therefore works in any position and does not require a liquid refill.

The gas sensing payload includes three Alphasense gas sensors (Alphasense, B4 type, Great Notley, Essex, UK) and one SprintIR CO_2_ sensor. The Alphasense sensors are electrochemical cells that operate in the amperometric mode and generate a current that is linearly proportional to the fractional volume of the measured gas. They are used to measure CO, NO and NO_2_ [15]. The SprintIR CO_2_ for CO_2_ concentration measurements is based on Non-Dispersive Infra-Red (NDIR) technology [16].

### 2.5. Payload Design and Telemetry

The UAV system architecture uses a radio modem to transmit real-time data including information on the three-dimensional location of the UAV and payload parameters to the ground station. The Arduino MEGA 2560 microcontroller (Arduino, Ivrea, Italy) transmits the data and was chosen over other devices such as the Raspberry Pi B+ microprocessor which might have both better hardware (e.g., memory) and software (e.g., operating system). However, such devices need more power and are slower. The Arduino is easier to use for its connectivity and programming, better speed for receiving and transmitting data, and lower power consumption. The Arduino can power all four gas sensors simultaneously.

### 2.6. Integration

**Figure 2:**
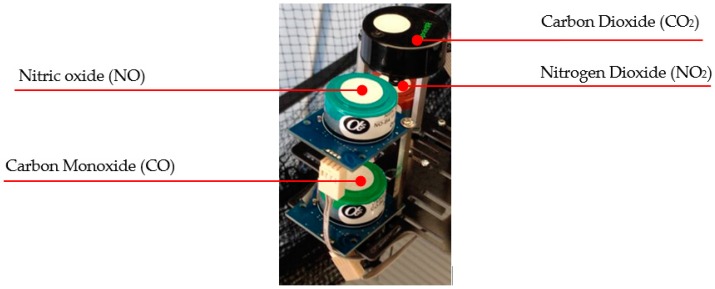
Queensland University of Technology (QUT) payload sensor system capable of measuring ${\;\mathrm{CO}}_{2},$ $\mathrm{CO},{\;\mathrm{NO}}_{2},\;\mathrm{NO}.$

The DiSCmini [17] can be easily integrated on the UAV as a small and lightweight monitor. However, careful positioning of the sensor to avoid possible issues with the aircraft center of gravity is needed. The custom made gas sensor payload (shown in Figure 2) includes the four gas sensors. Further details on each sensor are provided in Table S2 in the Supplementary Materials. The integration process resulted in a system that is capable of measuring PNCs and five gases simultaneously.

### 2.7. Gas Visualization Interface

The UAV system includes a ground control interface to visualize and store the data sent from the on-board sensors, in real-time. Figure S2 in the Supplementary Material presents a screenshot of this interface. 

The interface summarizes all data received from the gas sensors in a graph displayed on the right-hand side, while pressure, humidity and temperature measurements are displayed on the left-hand side. The instantaneous measurements are displayed at the bottom of the screen. The Ground Control Interface allows the user to store data using control buttons on top of the screen.

## 3. Experimental Design

**Figure 3:**
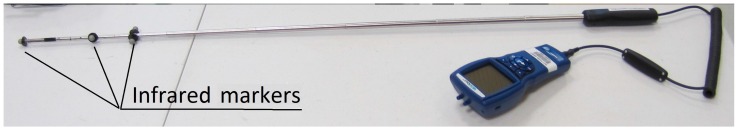
The Portable anemometer TSI, model 9565-P [19] with infrared targets to track and record the anemometer probe positon during Test 1 with the VICON system.

This research conducted two different tests to assess the optimal sensor intake location and the effectiveness of the sensors. For test 1, a professional standard anemometer (model 9565-P, TSI, Shoreview, MN, USA) was used to measure the air speed caused by the UAV propellers. The anemometer had 3% air velocity accuracy, 0–50 m/s measuring range and an accuracy of ±1.5% of reading and a resolution of 0.01 m/s (Figure 3) to create an airspeed map, to quantify the downwash and upwash effect of the S800 hexacopter. The portable anemometer was activated manually to store a reading, taken at 20 s intervals. Test 1 was designed to be conducted indoors to avoid any wind or external input that could have modified the UAV wash effect behavior.

As in Test 1, Test 2 was also conducted indoors and validated the functionality of the onboard sensors and their mounting location by measuring gas and particles of a plume emitted by a diesel engine as a source. Test 2 was further divided into three parts to record measurements inside, below and above the plume. 

### 3.1. Test 1

To solve the sensor mounting point issue so a boom was attached to the frame of the UAV to avoid turbulence and the air mixing effect of the propellers. The boom worked as both the mounting point for the gas sensors which work passively, and as sampling port for the DISCmini. The boom could extend alongside the UAV, above or even below it, using a cardan joint to fold it when the hexacopter lands. The air speed behavior was mapped along the UAV axes to determine which of the three available options was the most effective.

The experiment was conducted at the Australian Research Centre for Aerospace Automation (ARCAA) Indoor Flying Lab located in Brisbane, Queensland. The position of the anemometer was recorded by the VICON (Vicon Motion System Ltd., Oxford, UK) position mapping system an indoor tracking and localization system, used at ARCAA [18] to provide the position and orientation of an object inside the flight area [18]. The system is accurate to a sub millimeter level and provides 100 position readings per second. VICON provides the timestamp of each reading with an average position of 20 s at each interval to associate the air speed reading for each time interval.

**Figure 4:**
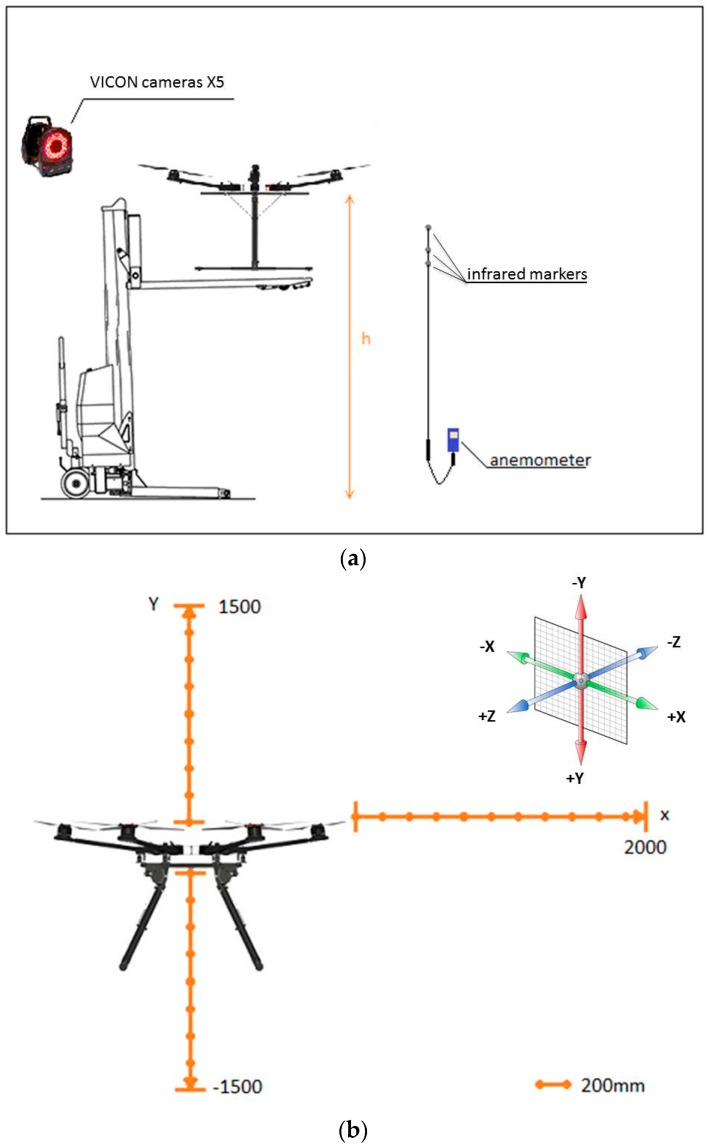
(**a**) Test 1 schematic representation; (**b**) Direction the anemometer probe was moved during the different experiments of Test 1.

An electrical forklift was used to fix the UAV, and provide an adjustable height mechanism (Figure 4a,b). The UAV was firmly attached to the forklift using zip ties, without blocking the space underneath the UAV so as to not affect propeller airflow (Figure S3 Supplementary Materials). 

Test 1 was divided into four experiments where measurements were taken along the Y axis, for both the negative, Experiment 1: −Y, and positive, Experiment 2: +Y, and along the X axis, including the horizontal, Experiment 3, and the vertical, Experiment 4, directions of the air flow. The UAV hexacopter has a symmetrical shape, and so it is assumed to have the same air speed profile along the Z and X axis.

**Figure 5:**
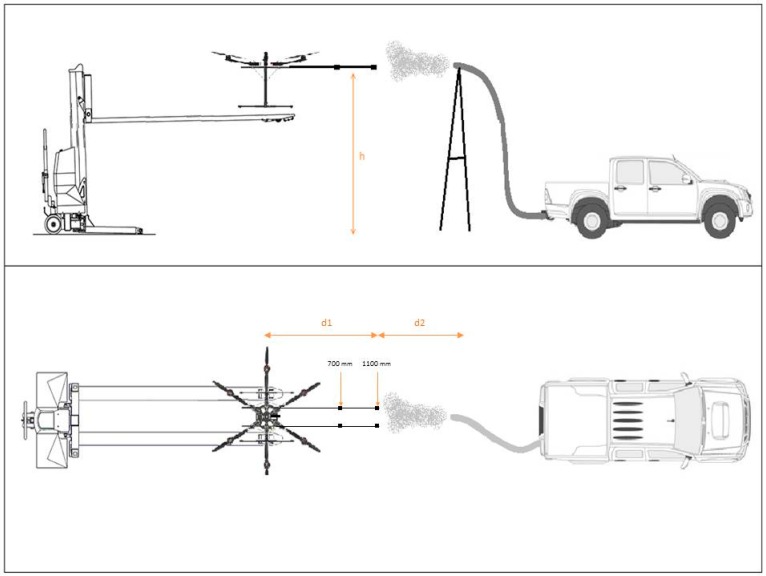
Test 2 schematic representation.

Figure 4a illustrates the set-up for Test 1 equipment and Figure 5b shows the direction of measurements taken every 100 mm. The maximum distance from the propellers where the sensors could be mounted was limited to 1200 mm, to maintain the UAV balance without the need of additional counterbalance weight at the other end. The total weight of the carbon tube that holds the sensors and the sensors themselves, is counterbalanced (offset) by the weight of the battery which is attached towards the back, underneath the UAV frame. 

Infrared detection markers were attached to the anemometer probe to record the anemometer position using the VICON system. Four infrared markers were placed on the UAV to record its position and as a reference (Figure 3 and Figure 4a). During each Test 1 experiment, one researcher was in charge of monitoring the marker positions, while another moved the anemometer along the three UAV axes, to ensure the measurements were taken correctly in terms of position and time. For both Experiments 1 and 2, the anemometer probe was moved from the reference, considered to be the center of the UAV at 0–1500 mm. During Experiments 3 and 4, the anemometer probe was moved along the X axis, from the closest point to the propellers, at 600 mm from the UAV center, to 1900 mm. During Test 1, the forklift to which UAV was attached was placed at a nominal height ‘h’ of three meters (h = 3000 mm).

### 3.2. Test 2

The overall aim of Test 2 was to quantify the propeller downwash effect on the sensor readings and to validate the UAV system in terms of its capability to collect data of gaseous and particle pollutants emitted by a pollution source. Test 2 compared the sensor measurements above, inside and below a plume emitted by the source. The sensors’ electrical signals were converted to concentrations using the manufacturer’s calibration values and equations. During Test 2, ground measurements of CO_2_ were also taken, while temperature and humidity were recorded by the onboard sensors. The two hangar doors were open in Test 2 to create an air current, and ensure that the plume from the combustion source was going in one direction. The UAV system was placed downwind to be in the plume. 

The aim of Test 2 was to answer the following questions:

(1) How does the status of the propeller (on/off) affect the measured gas and particle number concentrations?
(2) How does the position of the sensors (below, above or inside) affect the measured concentrations?
(3) How does the distance from the UAV center affect the measured gas and particle number concentrations?

The set-up for Test 2 is shown in Figure 5. The pollutant source was an Isuzu D-Max 4 × 4 Crew Cab Chassis (3.0 liter turbo diesel, in-line 4-cylinder, DOHC, 16-valve, with intercooled turbo charger, 380 nm, 130 kW, automatic). A 4000 mm heat-resistant aluminum flexible air ducting outlet tube was attached to the car exhaust. The pipe end was attached to the top of a ladder at a height of 2500 mm (Figure S4 Supplementary Materials). The indoor flight lab at ARCAA is a hangar with two specular doors. For this test, the D-Max was parked downwind with the engine running at the minimum RPM, to have a constant emission and to create a stable plume blowing towards the UAV system. As in Test 1, the UAV was fixed to the forklift in order have a precise and stable UAV system location. Based on the results of Test 1, two carbon fiber booms of 1000 mm each were attached to the UAV frame, along the X axis. The gas sensors were mounted on one boom, while the DISCmini nozzle was mounted on the other. The DISCmini was attached to the UAV frame. Two different positions along the carbon fiber booms were tested for the sensors and DISCmini nozzle at 700 and 1100 mm from the UAV center (distance ‘d1’ in Figure 5). These two positions were chosen because they offer the best compromise between minimum air speed and flight stability. Using a boom with the sensors mounted at a maximum distance of 1100 mm allows the UAV to fly, as the overall weight (sensors plus boom) is offset by the battery which is attached directly to the opposite side of the UAV frame. Figure S5 in the Supplementary Materials shows the sensors mounted onboard the UAV.

In Test 2, the UAV was placed in three different positions: 700 mm above the pipe exhaust, in front of the exhaust, and 700 mm below the exhaust. The distance of 700 mm was chosen as the position where the sensor recorded the same values of gases and PNCs of the background, confirming a reading outside the pollutant plume. The sensors were also moved along the boom in two positions to test how much the downwash effect affects the sensor readings. The distance between the sensor intakes and the plume source (exhaust pipe) was set to 700 mm and maintained for both sensor positions, respectively (d2 in Figure 5). The forklift was moved toward the source when the sensors were moved from 1100 mm (first mounting position for the sensors and DISCmini nozzle) to 700 mm along the boom. Test 2 comprised four different experiments which compared the sensor readings when the UAV system was in three different positions related to a plume from a pollutant source, and the sensor readings at two different mounting points. Experiment 1 was conducted with the propellers turned off, with the purpose of measuring the gases and PNC with no interference due to the propeller operation. During Experiments 2, 3 and 4, the propellers were turned on, the gas sensor distance from the UAV center varied (d1 in Figure 5) and measurements taken inside the pollutant plume at 2500 mm from the ground, outside the plume at 3200 mm (above the plume), and at 1800 mm from the ground (below the plume), respectively. The summary of these experiments is presented in Table 1.

## 4. Results

### 4.1. Test 1

**Figure 6:**
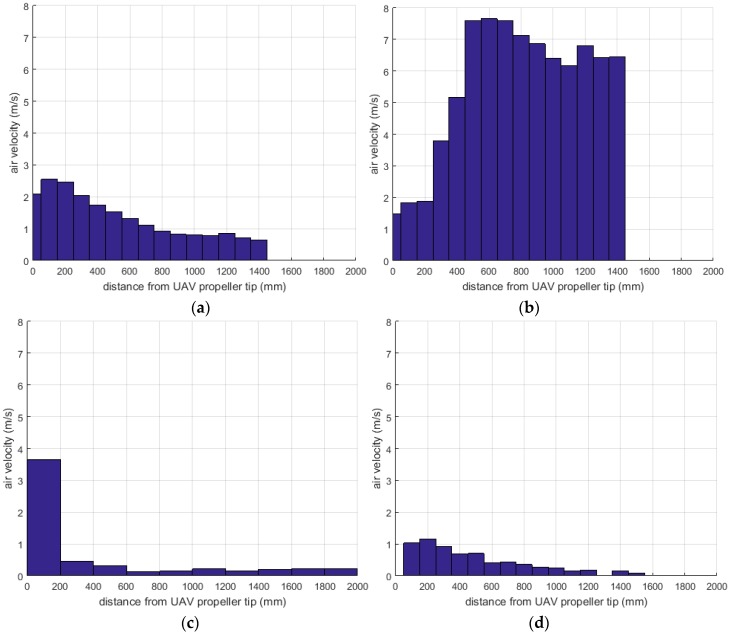
(**a**) Air speed along the Y axis of the UAV system, considering the positive direction component (+Y, above the UAV system); (**b**) Air speed along the Y axis of the UAV system, considering the negative direction of the values; (**c**) Air speed along the X axis of the UAV system—horizontal direction of air flow; (**d**) Air speed along the X axis of the UAV system—vertical direction of air flow.

Results of each of the three experiments, Experiment 1 (+Y), Experiment 2 (−Y), Experiment 3 and 4 (X), as part of Test 1, are shown in Figure 6a–d, respectively.

Figure 6a shows the air speed (m/s) measurements taken above the UAV during Test 1 Experiment 1. The data trend indicates that air speed decreases with distance from UAV to propeller tip, but plateaus at approximately 900 mm with an average speed of 0.9 m/s. Figure 6b shows the results of Experiment 2 for air speed readings below the UAV. The anemometer recorded an increase in air speed to 600 mm, starting from the center of the UAV and moving down. From that point, the airspeed decreased until 1200 mm and apart from a few fluctuations, stabilized with an average of 6.5 m/s. 

Figure 6c shows the result of Experiment 3 (horizontal direction of air flow), for air speed readings along the X axis. The hexacopter propellers do affect air flow, as expected, however air speed dropp quickly and become insignificant at distances over 700 mm. Figure 6d shows the result of Experiment 4 (vertical direction of air flow), for air speed readings along the same X axis, which demonstrated that the air speed decreased steadily at distances over 700 mm. 

The zero reference in both Figure 6c,d is just beyond the tip of the propeller. These results suggest that the propeller effect is more pronounced in the region closer to the propeller, and its effect lessens as distance increases.

**Figure 7:**
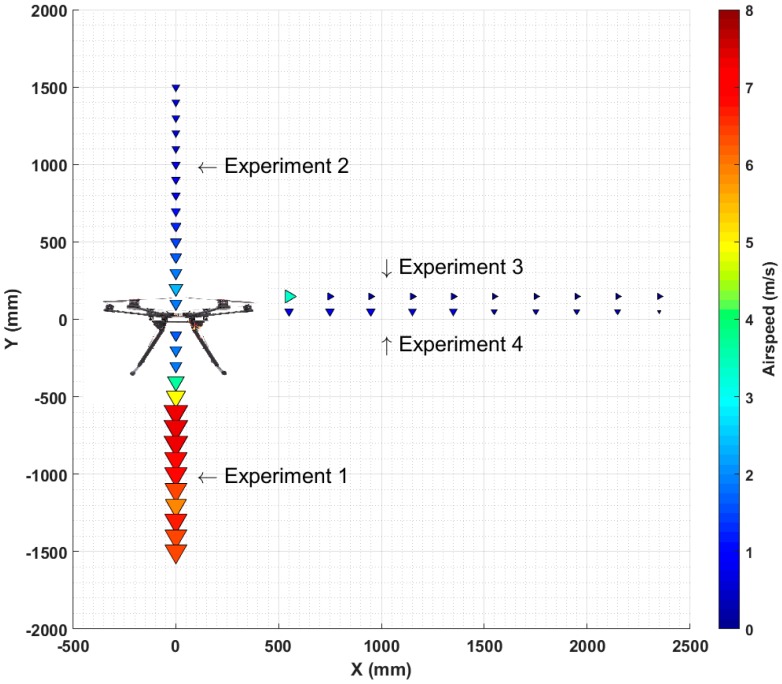
UAV system air velocity map.

The air speed data collected in all four experiments are presented in Figure 7 as an air speed map. Arrows of different size and color represent air speed. Arrows increase in size with an increase in air speed, while color moves from blue to red to indicate speed increment. 

Air speed measured above and below the UAV, along the Y axis (Experiment 1: +Y, Experiment 2: −Y) is represented with arrows pointing down. For example, air speed recorded during Experiment 2: −Y, at 500 mm underneath the UAV, was 5 m/s and increased to 7.5 m/s at 700 mm. Air speed measured along the X axis (Experiment 3: horizontal, Experiment 4: vertical) is represented with arrows pointing to the right of the UAV and down. For example, Experiment 3 showed that the air speed just beyond the propellers was about 4 m/s, and at 700 mm the air speed was less than 1 m/s. 

### 4.2. Test 2

**Figure 8:**
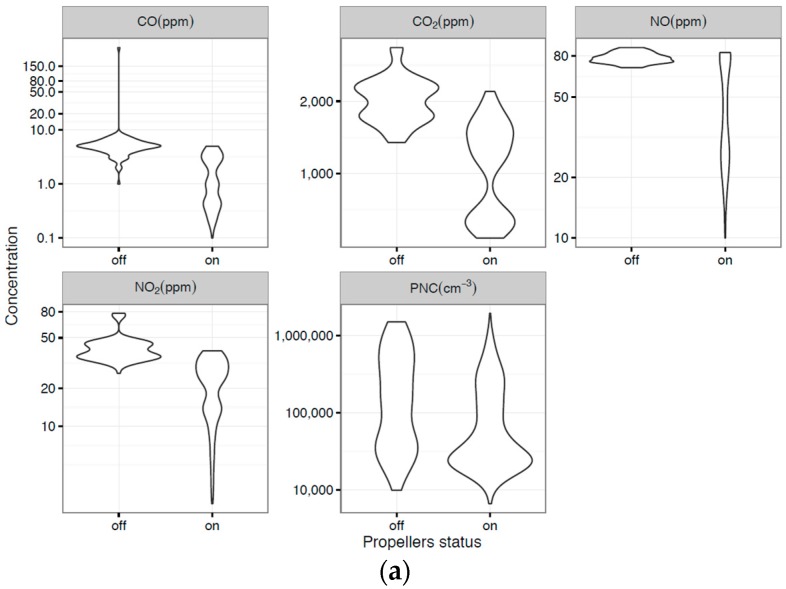

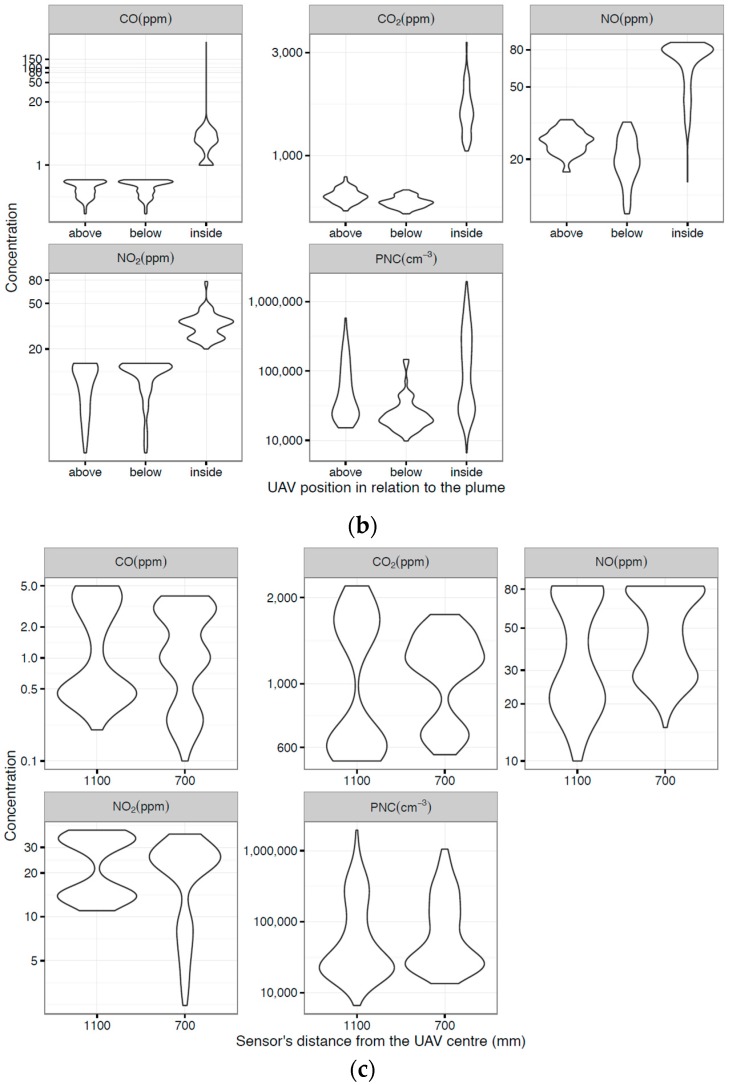
(**a**) Measured gas (ppm) and PNC (pt/cm^3^) concentrations with propellers turned off and on; (**b**) Measured gas (ppm) and PNC (pt/cm^3^) concentrations at three different positions relative to plume (inside, below and above); (**c**) Measured gas (ppm) and PNC (pt/cm^3^) concentrations measured at 1100 mm and 700 mm from the UAV center.

The distribution of Test 2 results is represented using violin plots (Figure 8a–c) where the width of each violin plot represents the density of data as measured for a particular concentration value. Violin plots are more informative than a plain box plot which only show a summary of mean/median and interquartile ranges for example [20]. Concentrations of measured pollutants were higher when the propellers were off (Figure 8a) and the UAV was inside the plume (Figure 8b).

Pollutant distribution under different conditions is presented as violin plots (Figure 8a–c) to show that measured concentrations were higher when the propeller was off (Figure 8a) and the UAV inside the plume (Figure 8b).

The Linear Regression model was developed from data collected when propellers were on, to predict the simultaneous effect of distance and position on the measured concentrations. The explanatory variables used are, distance from the UAV (in meters), and position (above, below, or inside (reference point).

The Linear Regression model was used to estimate the sensor position effects and their distance from the UAV center on measurements results. The model was structured as follows Equation (1):

(1) $$y_{i\;}=\;\beta_{0}+\beta_{1}p_{i}+\beta_{2}d_{i}$$

where: y is the measured concentration, i is the measurement number, p is a categorical variable indicating the position of the sensor (inside, above, or below), $\beta_{0}$ is the intercept, d is a continuous variable indicating the distance of the sensor from the center of the UAV in mm, $\beta_{1}$ and $\beta_{2}$ are the estimates. The Linear Regression model results identify the average changes in concentration with 95% confidence intervals. The inside-plume position was set as the reference position, in order to compare the effect of the positions below and above it.

Humidity and temperature measurements taken during the four experiments of Test 2 are presented in Figure S6a–g (Supplementary Material document). These measurements were recorded in real-time and show an increase in humidity when the UAV was inside the plume compared with the above and below positions. Temperature measurements were stable during all experiments with no significant variation between different UAV positions. Ground CO_2_ measurements were taken as a reference during Test 2, with results shown in Table 2.

## 5. Discussion

During Test 1, there was no air circulation within the room from air conditioning/filtering. Airspeed measurements in the room showed air currents were insignificant (<0.1 m/s) and stable before and after testing, with measurements taken during testing showing that the downwash caused by the UAV is mostly diffused within 3 m from the testing location. The effect of the airflow caused by the UAV on the measured parameters is expected to be negligible.

**Figure 9:**
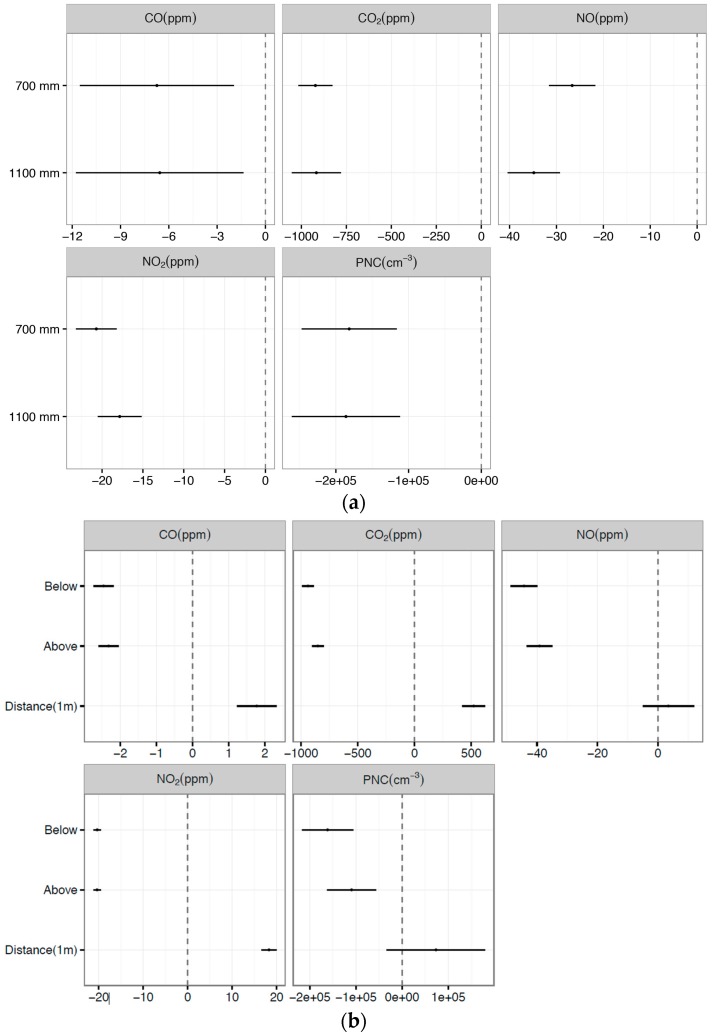
(**a**) Change in concentrations and 95% confidence intervals when propellers are on compared to off.; (**b**) Change in concentrations and 95% confidence intervals (per 1 m increase in sensor distance and position of sensor compared to inside the plume).

The UAV system air velocity map, obtained from Test 1 (Figure 7), showed that the best mounting point is along the X axis with a distance beyond the propellers between 1000 and 1200 mm. Figure 9a indicates that the concentrations of measured gases and PNC decrease significantly when the propellers are turned on. The 95% confidence interval (Figure 9a) represents the range where there is a 95% probability to establish the actual value of the pollutant concentrations measured. If the 95% confidence interval includes zero, then the estimate is not statistically significant, as was the case for the change of distance for NO, and PNC. However, the decrease of PNC is so small that it lies within the level of instrumental uncertainty. The decrease in measured concentration when the propellers are on is simply due to the dispersion of the plume. 

The effect of position (below or above) compared to inside the plume is negative, which means that the concentration of both the gases and PN is lower when the UAV is above or below the plume compared to when the UAV is inside the plume. Figure 9b and the Linear Regression model results (Equation (1)) indicate that the measured concentration of gases and PNC changes per 1 meter increase with sensor distance for all pollutants, except PNC and NO. Furthermore, the measured concentrations decrease significantly for all pollutants when the sensor is located above or below, compared with inside the plume (Figure 9b). Test 2 results demonstrate that UAV propellers cause a dispersion effect, as shown by a decrease of gas concentration as read by the sensors when mounted closer to the UAV center. In fact, moving the sensors farther from the UAV center reduces the dispersion effect. The difference in value is evident by comparing the results in Figure 9a,b where their significance is visualized by confidence intervals. During Test 2, it was found that while the response time of the sensors was somewhat decreased, it was still an acceptable response, as the sensors are designed to work passively. The main reason for not using active sampling, however, is because the additional weight added to the end of the boom began to offset the amount of thrust available to counterbalance the additional weight created by using such a set-up. It was found that the alphasense sensors had an average response time in the order of 3–6 s, with the added airflow caused by the propeller wash actually helping with the response time by cycling the air around the entire system. Test 2 also demonstrated the capability of the UAV system to collect and transmit data with the position of the UAV system inside and outside a pollutant plume. This finding is important when the aim is to stay inside the plume. The UAV system should be flown in a way that the sensors are inside the plume and as far from the UAV center as possible. This positioning is confirmed by an increase in relative humidity while the temperature is stable due to the quick cooling of the outdoor air when it mixes with the plume. 

The ISUZU D-Max was the pollution source used to validate the UAV system presented in this research. Real world applications include measurement of small plumes emitted by a cargo or cruise ship.

## 6. Conclusions

Monitoring air quality using small sensors onboard a UAV is very complicated, not only for constraints such as power consumption, weight and propeller effect, but also because the choice of sensors depends on the pollution source being measured.

Real-test data collection must consider the nature of the mission to evaluate the best compromise between feasibility to fly and accuracy of data. That is, a small plume emitted by a stationary (industrial stacks) or a moving (ships, in-land vehicles) source. The UAV system presented in this research has been designed for the purpose of combustion source emission measurements. These types of missions normally comprise low speed and linear flight paths, such as taking off, flying to and hovering in the sampling position, and landing, which means the UAV system should be flown in a way where the sensors are inside the plume and as far from the UAV center as feasible.

An alternative is required when there is strong wind or where the flight path involves fast, frequent and steep changes of direction, such a chasing a fast moving source. This action could damage the payload, which makes positioning of the sensor closer to the UAV system center preferable, even though the error of the reading is higher. This research presents for the first time a comprehensive analysis of what needs to be done to optimize the development of UAV systems so they are more accurate in real time applications. The Linear Regression model is an example of a model that could be used for further studies and a similar approach could be used by other researchers who use different UAV platforms where the focus could be not only on the integration of sensors onboard UAV, but also on quantitative data collection. It is feasible to integrate air quality sensors onboard a UAV system to measure gaseous and particle pollutants in time and space. Further work will focus on real-world applications and the analysis of near-real time data to help atmospheric modelling software development and flight path planning algorithms.

## Figures and Tables

**Table 1:** Summary of Test 2 Experiments 1–4.

	Propeller Status	UAV Position, UAV Height from the Ground (mm)	Sensor Intake Position; Distance from UAV Center (mm)	Sensor Position; Distance from Exhaust (mm)
Experiment 1	Off	Inside the plume, 2500	-	700
Experiment 2a	On	Inside the plume, 2500	1100	700
Experiment 2b	On	Inside the plume, 2500	700	700
Experiment 3a	On	Above the plume, 3200	1100	700
Experiment 3b	On	Above the plume, 3200	700	700
Experiment 4a	On	Below the plume, 1800	1100	700
Experiment 4b	On	Below the plume, 1800	700	700

**Table 2:** Test 2 ground measurements summary.

Experiment	1	2a	2b	3a	3b	4a	4b
CO_2_ (ppm)	558	548	546	580	524	554	576

## Supplementary Materials

The Supplementary Materials are available online at http://www.mdpi.com/1424-8220/16/12/2202/s1.

## Abbreviations

The following abbreviations are used in this manuscript:

UAV	Unmanned aerial vehicle
UAS	Unmanned aerial system
PM	Particulate matter
VOC	Volatile organic compounds
UFP	Ultrafine particles
FAA	Federal Aviation Authority of the U.S.
CASA	Civil Aviation Safety Australia
ARCAA	Australian Research Center for Aerospace Automation
PNC	Particle Number Concentration
