# Supplementary material for: Development and Validation of a UAV Based System for Air Pollution Measurements
Source: Sensors (Basel). 2016 Dec 21;16(12):2202. doi: 10.3390/s16122202 (PMC5191180; doi:10.3390/s16122202)
Supplement: Supplementary file 1 [file sensors-16-02202-s001.pdf]

Supplementary Materials: Development and Validation of a UAV Based System for Air Pollution Measurements

Tommaso Francesco Villa, Farhad Salimi, Kye Morton, Lidia Morawska and Felipe Gonzalez

Table S1. DISCmini technical specifications.

**Mean Particle Size**	**10 to 300 nm (Modal Diameter)**
Particles counted	10 to 700 nm
Particle concentration	Detectable particle concentrations depend on particle size and averaging time. Typical values are given below. 20 nm: 2E3 to 1E6 pt/ccm 100 nm: 5E2 to 5E5 pt/ccm
Accuracy	±30% in size and number typical; ±5E2/ccm absolute in number
Flow rate	1.0 L/min ± 0.1 L/min
Pressure	800 to 1100 mbar abs ambient; Δp max. at inlet: ±20 mbar
Temperature	+10 to +30 °C; relative humidity
Time resolution	1 s
Dimensions	120 mm× 80 mm× 40 mm
Weight	0.7 kg
Power requirements	The battery charger is compatible with the any 100 to120 v or 200 to 240 v 50/60 Hz AC wall outlet
Battery lifetime	8 h typical; varies with ambient temperature. Charging time 2 to 4 h depending on charger and status of battery.

Table S2. Sensor features of the customized payload. Source: Sensor datasheets. nA: nanoampere; ppm: part per million; ppb: part per billion; FS: full scale. Table S2 reports the six different gas sensors which were integrated and tested as part of the hexacopter payload. However, the SO_2 and O_3 sensors did not perform, giving unrealistic values and therefore the data collected by these sensors were not considered.

Sensor	Sensitivity (nA/ppm at 2 ppm)	Response Time	Zero current (nA in Zero Air at 20 °C)	Noise ±2 Standard Deviations (ppb Equivalent)	Range (ppm)	Linearity	Over Gas Limit (Maximum ppm for Stable Response to Gas Pulse)
CO	CO 420 to 650	t90 (s) from zero to 10 ppm CO < 25	+30 to −130	4	ppm limit of performance warranty 1000	ppb CO error at full scale, linear at zero, 500 ppm CO 20 to 35	2000
SO2	SO2 275 to 475	t90 (s) from zero to 2 ppm SO2 < 30	−80 to +80	5	ppm limit of performance warranty 100	ppb error at 100 ppm SO2 , linear at zero and 10 ppm SO2 0 to −2	200
NO	NO 500 to 850	t90 (s) from zero to 2 ppm NO < 45	30 to 140	15	ppm NO limit of performance warranty 20	ppb error at full scale, linear at zero and 5 ppm NO < ±1	50
NO2	NO2 −223 to −470	t90 (s) from zero to 2 ppm NO2 < 35	0 to 70	15	ppm NO2 limit of performance warranty 20	ppm error at full scale, linear at zero and 20 ppm NO2 < ±0.5	50
O3	O3 −225 to −525	t90 (s) from zero to 1 ppm O3 < 35	−50 to 70	15	ppm O3 limit of performance warranty 20	ppm error at full scale, linear at zero and 20 ppm O3 < ±0.5	50
CO2	0 to 10,000 ppm	Response Time • 30 s to 2 min (user Configurable) 3 • Reading refreshed twice per second 3 Warm-up Time •<10 s		Low noise measurement (<10 ppm)		Non Linearity • <1% of FS	

Figure S1 shows both the hardware and software of the UAV system, including the ground control station, with the connection types between the different UAV components highlighted. Dark lines indicate physical connections, i.e., for motors and props, while red arrows show the human input into the platform. The data link among the onborad components is represented by orange arrows. The dash-lines linking the UAV platform and payload components with those of the ground control station indicate wireless connections.

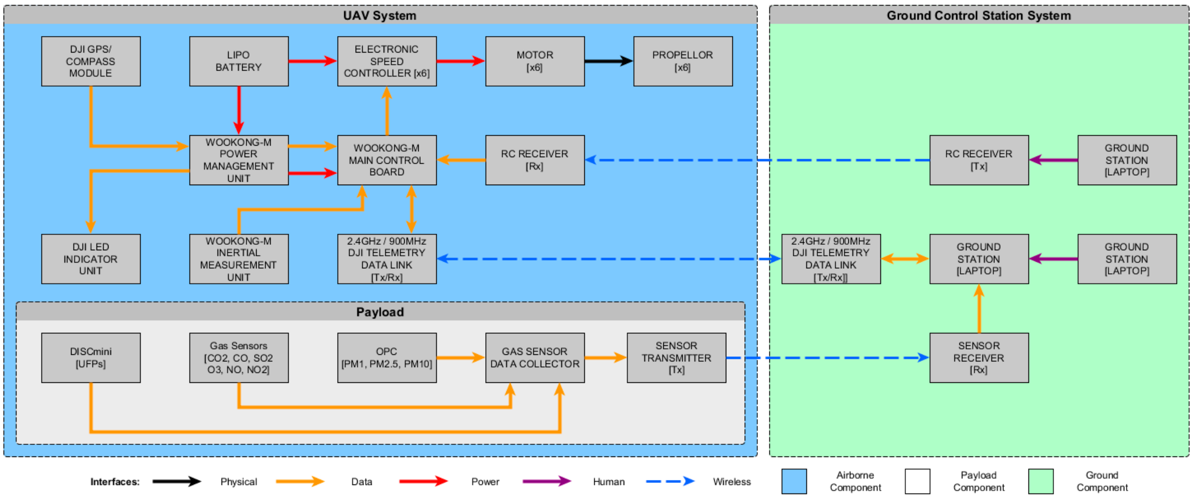

Figure S1. UAV and ground base system architecture.

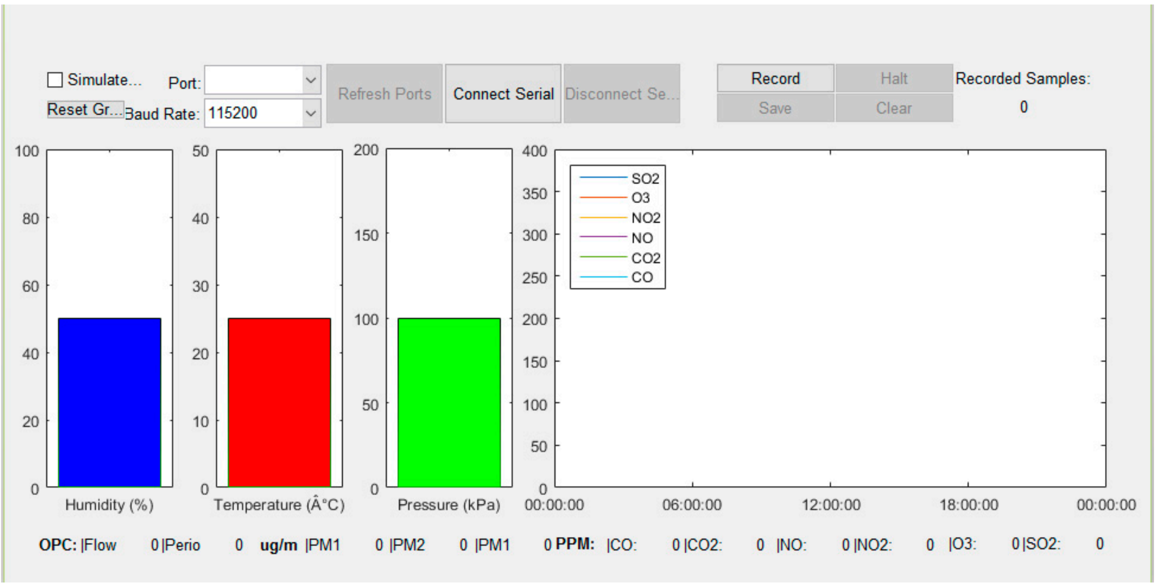

Figure S2. Ground Control Interface screenshot.

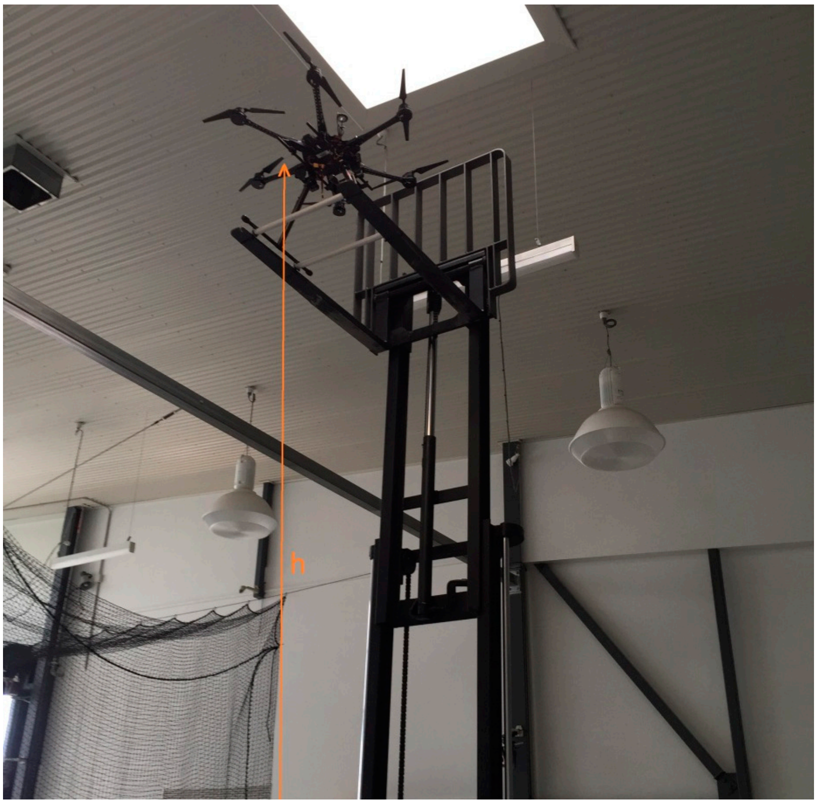

Figure S3. The S800 EVO fixed to the forklift inside the flight area.

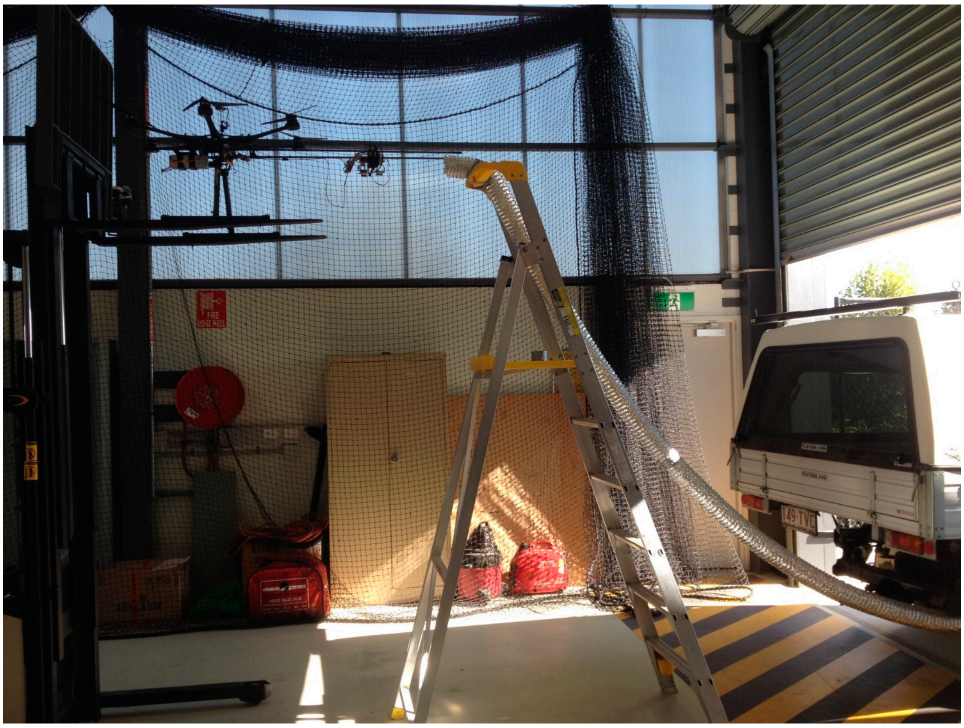

Figure S4. Experimental set up for Test 2.

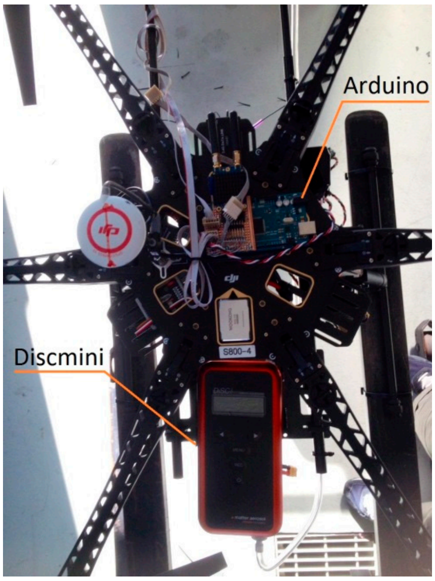

Figure S5. Discmini sensor mounted on S800 UAV.

Appendix A

- ***Experiment 1***

Humidity and Temperature On-Board the UAV

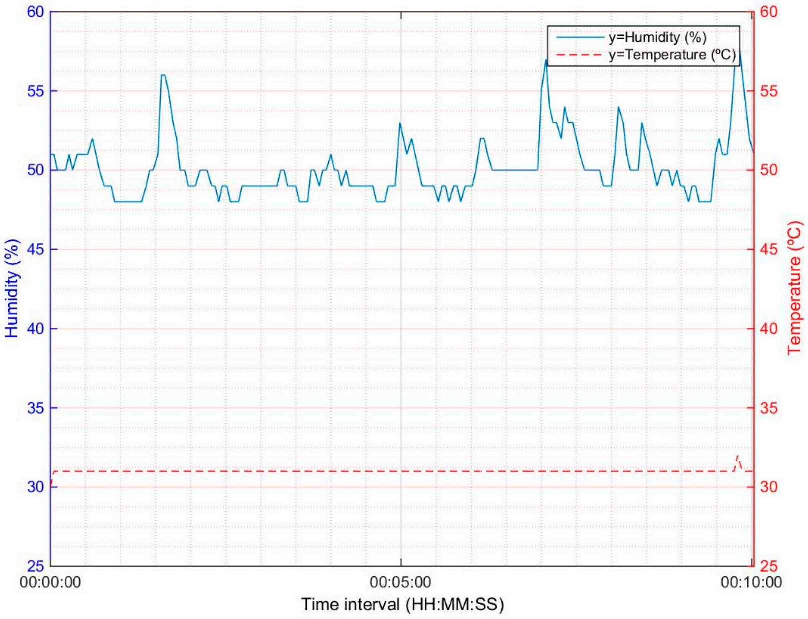

Figure S6a. Humidity and temperature measurements in experiment 1 with the sensors at 1100 mm from the centre of the UAV, the propellers turned off and the UAV at 2500 mm from the ground, in front of the exhaust.

- ***Experiment 2a***

Humidity and Temperature On-Board the UAV

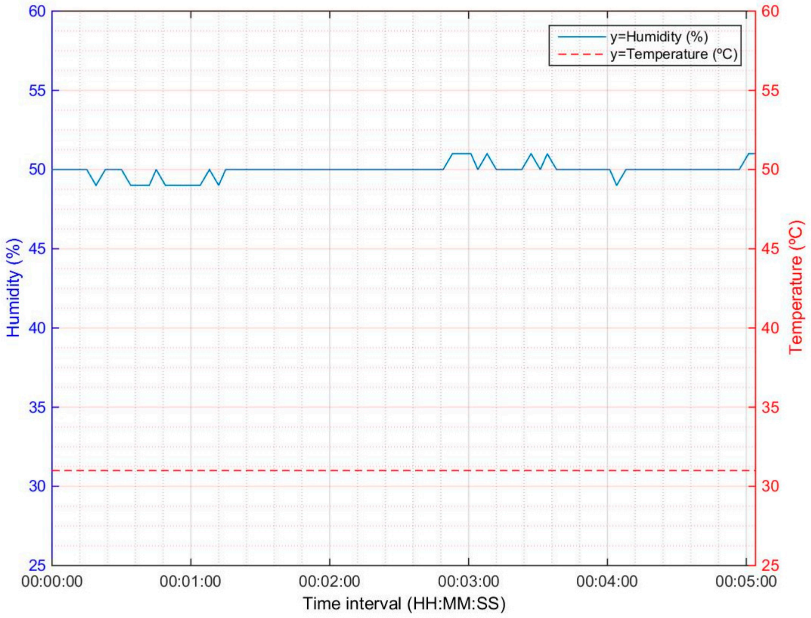

Figure S6b. Humidity and temperature measurements in experiment 2a, inside the plume. The sensors position was at 1100 mm from the centre of the UAV, the propellers turned on and the UAV at 2500 mm from the ground, in front of the exhaust.

- ***Experiment 2b***

Humidity and Temperature On-Board the UAV

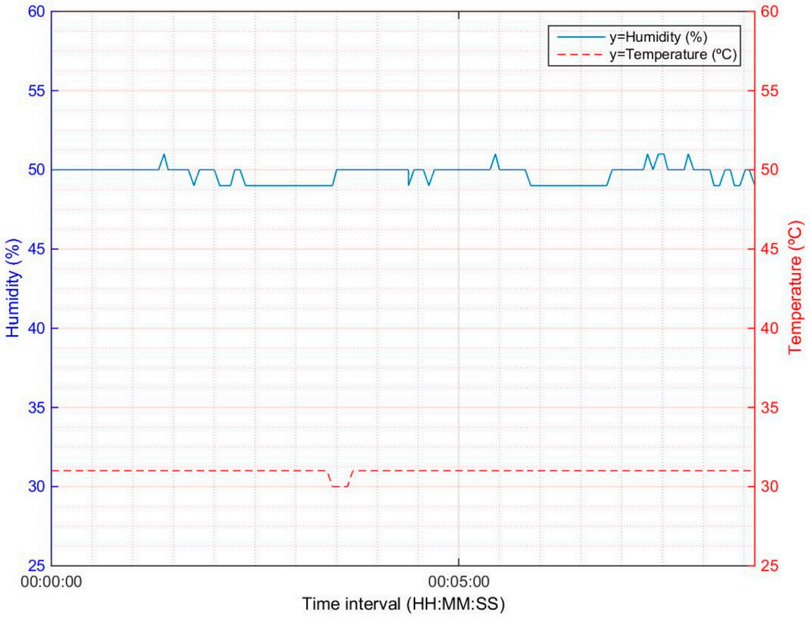

Figure S6c. Humidity and temperature measurements in experiment 2b, inside the plume.The sensors position was at 700 mm from the centre of the UAV, the propellers turned on and the UAV at 2500 mm from the ground, in front of the exhaust.

- ***Experiment 3a***

Humidity and Temperature On-Board the UAV

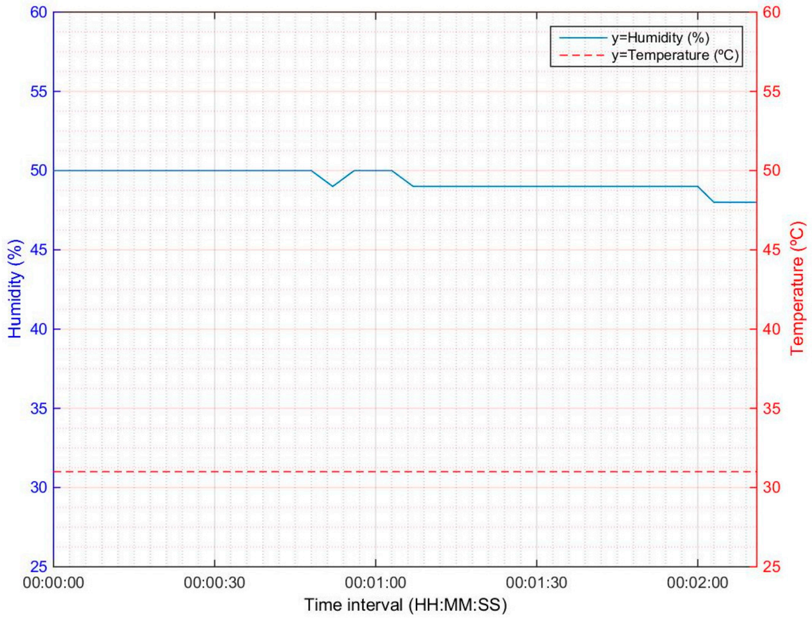

Figure S6d. Humidity and temperature measurements in experiment 3a, above the plume. The sensors position was at 1100 mm from the centre of the UAV, the propellers turned on and the UAV at 3200 mm from the ground, 700 mm above the exhaust.

- ***Experiment 3b***

Humidity and Temperature On-Board the UAV

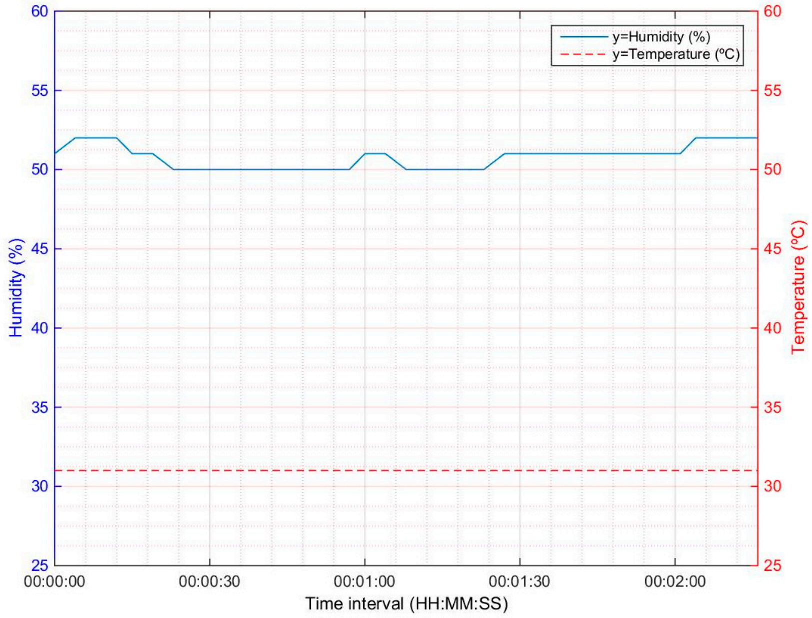

Figure S6e. Humidity and temperature measurements in experiment 3b, above the plume. The sensors position was at 700 mm from the centre of the UAV, the propellers turned on and the UAV at 3200 mm from the ground, 700 mm above the exhaust.

- ***Experiment 4a***

Humidity and Temperature On-Board the UAV

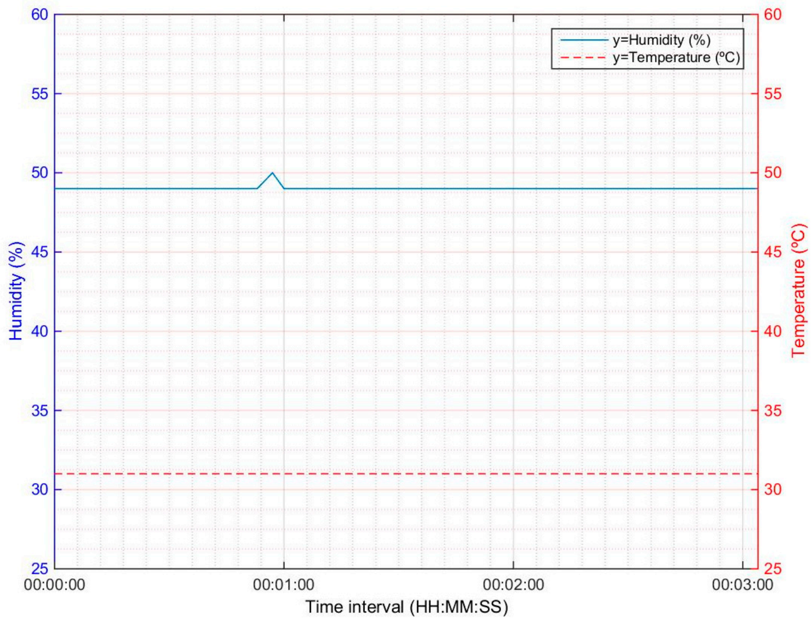

Figure S6f. Humidity and temperature measurements in experiment 4a, below the plume. The sensors position was at 1100 mm from the centre of the UAV, the propellers turned on and the UAV at 1800 mm from the ground, 700 mm below the exhaust.

- ***Experiment 4b***

Humidity and temperature on-board the UAV

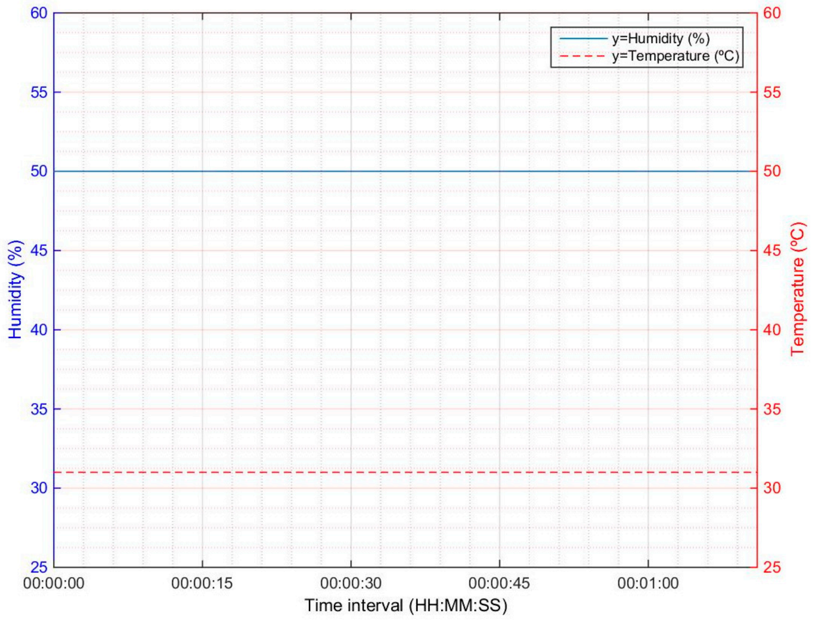

Figure S6g. Humidity and temperature measurements in experiment 4b, below the plume. The sensors position was at 700 mm from the centre of the UAV, the propellers turned on and the UAV at 1800 mm from the ground, 700 mm below the exhaust.
